# Ventral Tegmental Area Projection Regulates Glutamatergic Transmission in Nucleus Accumbens

**DOI:** 10.1038/s41598-019-55007-y

**Published:** 2019-12-05

**Authors:** Jun Yu, Masago Ishikawa, Junshi Wang, Oliver M. Schlüter, Susan R. Sesack, Yan Dong

**Affiliations:** Department of Neuroscience, University of Pittsburgh, Pittsburgh, PA 15260 USA

**Keywords:** Neuroscience, Cellular neuroscience

## Abstract

The ventral tegmental area (VTA) projection to the nucleus accumbens shell (NAcSh) regulates NAcSh-mediated motivated behaviors in part by modulating the glutamatergic inputs. This modulation is likely to be mediated by multiple substances released from VTA axons, whose phenotypic diversity is illustrated here by ultrastructural examination. Furthermore, we show in mouse brain slices that a brief optogenetic stimulation of VTA-to-NAc projection induced a transient inhibition of excitatory postsynaptic currents (EPSCs) in NAcSh principal medium spiny neurons (MSNs). This inhibition was not accompanied by detectable alterations in presynaptic release properties of electrically-evoked EPSCs, suggesting a postsynaptic mechanism. The VTA projection to the NAcSh releases dopamine, GABA and glutamate, and induces the release of other neuronal substrates that are capable of regulating synaptic transmission. However, pharmacological inhibition of dopamine D1 or D2 receptors, GABAA or GABAB receptors, NMDA receptors, P2Y1 ATP receptors, metabotropic glutamate receptor 5, and TRP channels did not prevent this short-term inhibition. These results suggest that an unknown mechanism mediates this form of short-term plasticity induced by the VTA-to-NAc projection.

## Introduction

The nucleus accumbens (NAc) shell (Sh) is a key brain region that receives and integrates convergent emotional and motivational signals that aid in regulating behavioral output^1,2^. These signals are thought to be mediated, in part, by glutamatergic inputs from several limbic and paralimbic brain regions that make monosynaptic connections to principal medium spiny neurons (MSNs), the only neuronal output from the NAcSh^1–3^. NAcSh MSNs can be  roughly divided into two subpopulations, one expressing dopamine D1 receptors and the other expressing D2 receptors, with a potential third population expressing both D1 and D2 receptors^4^. D1 and D2 NAcSh MSNs are preferentially involved in different neural circuits, activation of which often results in different behavioral consequences^4–6^. D1 and D2 NAcSh MSNs receive similar but slightly biased intensity of glutamatergic inputs, and both are activated during emotional and motivational responses^4,5,7,8^.

NAc-mediated emotional and motivational responses are critically regulated by the ascending projection from the ventral tegmental area (VTA)^9,10^. As the backbone of the mesolimbic dopamine system, the VTA-to-NAc projection regulates glutamatergic inputs to MSNs by releasing dopamine, glutamate, GABA, BDNF, and other signaling factors^11–15^. During the performance of motivated behaviors, different cell populations in the VTA are activated within the same time block, presumably leading to co-release of all these molecular regulators^15,16^. Although the impact of each of these VTA-originating regulators on NAc glutamatergic synaptic transmission has been examined pharmacologically, the impact of potential co-release of these regulators following activation of the VTA-to-NAc projection remains unclear.

In the current study, we optogenetically stimulated the VTA-to-NAc projection in mouse *ex vivo* brain slices, which resulted in a transient inhibition of electrically-evoked excitatory synaptic currents (EPSCs) in both NAcSh D1 and D2 MSNs. This inhibition remained intact in the presence of an antagonist cocktail that inhibited GABA_A_ and GABA_B_ receptors, cannabinoid receptor type 1, NMDA receptors, dopamine D1 and D2 receptors, ATP receptors, metabotropic glutamate receptor 5, as well as TRP channels. These results suggest that an unknown mechanism utilized by the VTA-to-NAc projection transiently inhibits the glutamatergic synaptic transmission to NAcSh MSNs.

## Results

**Figure 1:**
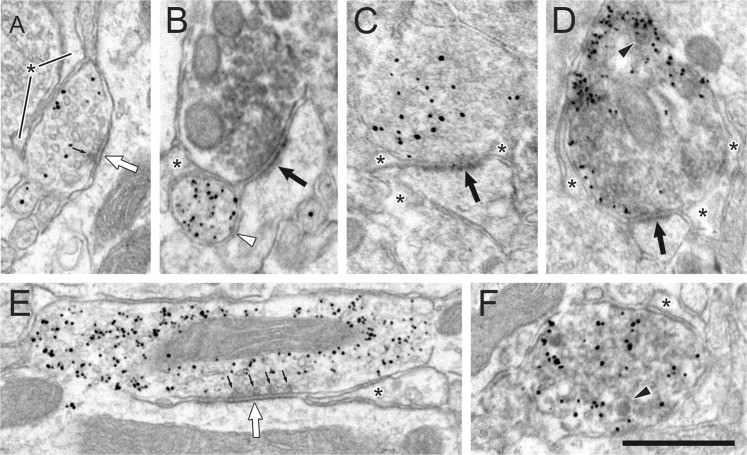
Electron micrographic images of the VTA projection to the NAc in the mouse. Silver-enhanced immunogold for eGFP anterogradely transported from the VTA is found in axons with a variety of morphological phenotypes. Panels (A,B) show axons with features characteristic of dopamine projections. The varicosity in (**A**) exhibits a single presynaptic dense projection (small black arrow) and forms a short symmetric synapse (large white arrow) onto an unlabeled dendrite. The varicosity in (**B**) is apposed (white arrowhead) to the neck of an unlabeled dendritic spine that receives an asymmetric synapse on its head (large black arrow) from an axon containing immunoperoxidase for vGlut2. Panels (C,D) depict axons with the morphological features of glutamate projections. Both form asymmetric synapses (large black arrows) onto unlabeled dendritic spines. In (**D**), the axon is dually-labeled for the eGFP tracer and for vGlut2 and also exhibits a dense-core vesicle (black arrowhead). Panel (E) shows a heavily labeled axon forming a symmetric synapse (large white arrow) onto an unlabeled dendrite. The large size of this axon, the extensive synaptic length, and the presence of multiple presynaptic dense projections (small black arrows) suggest a GABAergic phenotype. Panel (F) illustrates an axon varicosity dually-labeled for eGFP and vGlut2 and containing a dense-core vesicle (black arrowhead). Besides glutamate, other transmitters that might be contained in this varicosity are unknown, because the axon, like many VTA projections, does not form a synapse in single sections. In all panels, axons projecting from the VTA to the NAc lie in contact with astrocytic processes (asterisks). Scale bar in (**F**), 0.6 µm.

The VTA projection to the NAc is thought to release a variety of neurotransmitters and neuronal factors. Many of the studies supporting this view were performed in rats. To verify the phenotypic diversity of this projection at the ultrastructural level in the mouse, we injected enhanced GPF (eGFP) into the VTA and examined anterograde transport to the NAcSh. In the electron microscope, silver-enhanced immunogold labeling for eGFP transported from the VTA was found almost exclusively in axon varicosities, and these exhibited a variety of morphological phenotypes (Fig. 1). Dopamine-like axons were suggested by relatively short or absent symmetric-type synapses^17^ targeting dendritic shafts and the necks of dendritic spines^18–20^ (Fig. 1A,B). Other axons forming longer, more pronounced synapses were suggestive of GABAergic projections from the VTA^21,22^ (Fig. 1E). The presence of glutamate in some VTA to NAc axons was indicated by the formation of synapses of asymmetric type^17^ onto dendritic spines (Fig. 1C); some of the axons with this morphology also contained immunoperoxidase labeling for the vesicular glutamate transporter type 2^23,24^ (vGlut2; Fig. 1D). The content of dense-core vesicles in some VTA to NAc axons (Fig. 1D,F) is consistent with the presence of peptide co-transmitters in this pathway^25,26^. The axons exhibiting immunolabeling for eGFP transported from the VTA were often found in contact with astrocytic processes in the NAc (Fig. 1).

**Figure 2:**
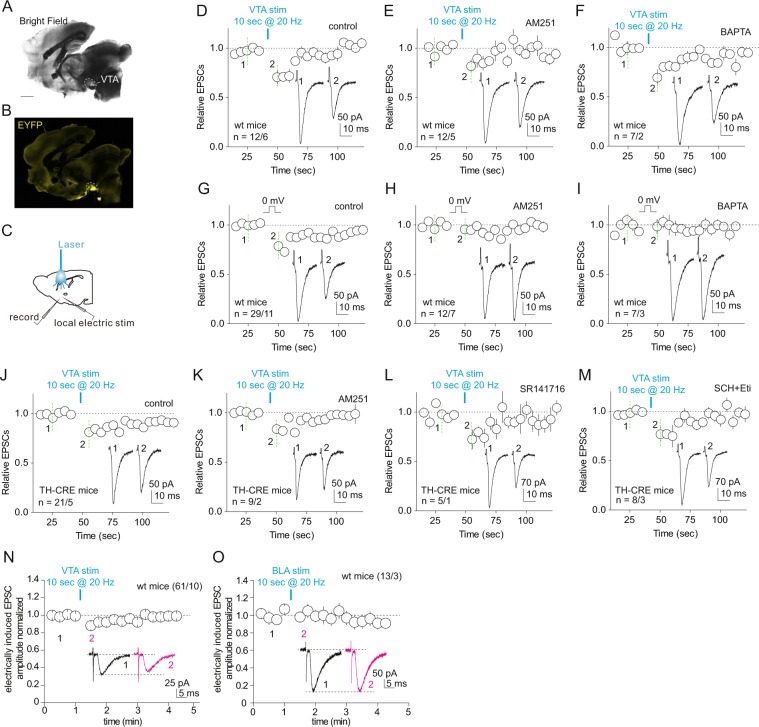
VTA projection modulates EPSCs in NAcSh MSNs. (**A**,**B**) Example images from the bright field (**A**) and EYFP (**B**) channels showing expression of ChR2 in the VTA within a sagittal slice (ML 0.35 mm). Scale bar, 1 mm. (**C**) Diagram showing recording of electrically-evoked EPSCs in NAcSh MSNs evoked by electrical stimulation in response to optogenetic stimulation of the VTA-to-NAc projection. (**D–F**) Summaries showing that optogenetic activation of VTA-to-NAc projection induced a short-term inhibition of electrically-evoked EPSCs in NAc MSNs from wildtype mice (**D**), and this effect was not prevented by bath-application of the CB1-selective antagonist AM251 (**E**) or intracellular infusion of the calcium chelator BAPTA (**F**). (**G**–**I**) DSE induced by brief depolarization (0 mV for 10 sec) in NAc MSNs from wildtype mice (**G**) was prevented by bath application of AM251 (**H**) or intra-cellular perfusion of BAPTA (**I**). (**J**–**M**) In TH-CRE mice in which the VTA dopaminergic projection was selectively stimulated, the short-term inhibition was similarly induced in NAc MSNs (**J**), and this effect was not prevented by bath application of AM251 (**K**), SR141716 (**L**), or a combined application of SCH23390 (SCH) and eticlopride (Eti) (**M**). (**N**,**O**) Summaries showing that activation of the VTA-to-NAc projection (**N**), but not the BLA-to-NAc projection (**O**), induced a short-term inhibition of the electrically-evoked EPSCs in NAcSh MSNs.

To examine the impact of activation of the VTA-to-NAc projection on NAc excitatory synaptic transmission, we bilaterally injected channel rhodopsin 2 (ChR2)-expressing adeno-associated virus 2 into the VTA of wildtype or transgenic mice. Five to six weeks later, we prepared sagittal slices containing both the NAc and VTA projection fibers (Fig. 2A). Expression of ChR2-YFP was visually detected in the VTA as well as VTA projection fibers in the NAcSh (Fig. 2B). We made whole-cell voltage-clamp recordings from NAcSh MSNs and recorded EPSCs evoked by an electrical stimulator placed ~200 μm from the recorded neurons (Fig. 2C). These EPSCs were locally evoked by electrical stimulation at fixed frequencies (e.g., once either 5 or 7.5 sec) continuously throughout the experiments, and were thus operationally defined as “electrically-evoked EPSCs” to differentiate them from optogenetically-evoked EPSCs in latter experiments. 50 μM GABA_A_ receptor antagonist picrotoxin was included in all recordings. After achieving a stable baseline of electrically-evoked EPSCs, we applied a train of laser stimulation (473-nm, 0.5–2 W to the slice surface; 1-ms pulse duration, 20 Hz over 10 sec), which resulted in a transient inhibition of the electrically-evoked EPSCs in NAcSh MSNs (relative EPSCs over the 30-sec period after optogenetic stimulation: 0.81 ± 0.03, p < 0.01, n/m = 12/6, paired t-test; Fig. 2D), with a pattern similar to depolarization-induced suppression of excitatory synaptic transmission (DSE)^27^.

DSE is typically triggered by a brief but sustained postsynaptic depolarization, which, in turn, induces release of endocannabinoids that activate presynaptically located cannabinoid receptor 1 (CB1) to reduce release probability during excitatory synaptic transmission^27^. The nonselective viral vector results in expression of ChR2 in all infected VTA neurons, including both dopaminergic and GABAergic neurons^28,29^. As such, optogenetic stimulation of VTA projections under this experimental condition not only evokes release of dopamine, but also glutamate and GABA that can acutely shift the membrane potential of NAc MSNs^28,29^. To test whether this VTA-induced short-term inhibition is indeed a DSE-like effect induced by depolarization of NAc MSNS, we performed the same experiment in the presence of the CB1-selective antagonist AM251 (2.5 μM), or included a calcium ion chelator BAPTA in the recording electrodes. Neither of these manipulations prevented this VTA projection-induced transient inhibition (relative EPSCs in AM251: 0.89 ± 0.05, p = 0.03, 12/5; in BAPTA: 0.79 ± 0.03, p < 0.01, 7/2; Fig. 2E,F), although either of these manipulations was sufficient to prevent the classic DSE induced in NAc MSNs (relative EPSCs in control bath: 0.83 ± 0.02, p < 0.01, 29/11; in AM251: 0.95 ± 0.03, p = 0.10, n/m = 12/7; in BAPTA: 0.98 ± 0.05, p = 0.70, n/m = 7/3; Fig. 2G–I). Thus, the potential depolarization induced by optogenetic stimulation of VTA projection is not likely involved in this transient inhibition.

Both dopaminergic and GABAergic neurons in the VTA project to the NAc. Our subsequent results show that stimulation of projections from VTA dopaminergic neurons is sufficient to transiently inhibit electrically-evoked EPSCs in NAc MSNs. Specifically, we injected AAV2-flexed ChR2R into the VTA of TH-IRES-Cre mice (B6.129 × 1-Thtm1(cre)Te/Kieg, the European Mouse Mutant Archive)^30^, and prepared NAc-containing brain slices 4–6 weeks later. In this preparation, transient stimulation of VTA dopaminergic projections induced a similar short-term inhibition of the excitatory synaptic transmission to NAc MSNs, which was not prevented by CB1-selective antagonists AM251 (2.5 μM) or SR141716 (2.5 μM), or dopamine D1- and D2 receptor-selective antagonists SCH23390 (10 μM) and eticlopride (8 μM) (relative EPSCs in control bath: 0.86 ± 0.04, p < 0.01, 21/5; in AM251: 0.89 ± 0.03, p < 0.01, n/m = 9/2; in SR141716: 0.81 ± 0.05, p = 0.02, n/m = 5/1; in SCH23390 and eticlopride: 0.84 ± 0.06, p = 0.025, n/m = 8/3; Fig. 2J–M). Thus, although the selective activation of the dopaminergic VTA projection induced a short-term inhibition of excitatory synaptic transmission in the NAc, this effect was not likely mediated by dopamine receptors.

A legitimate concern was whether such a transient and relatively weak effect might have resulted from optogenetic manipulation-associated heat generation and tissue damage^31^. Although the parameters of our optogenetic stimulation were rather moderate and should not have produced such tissue damage, we still performed a control experiment. We virally expressed ChR2 or a variant of rhodopsin Chrimson^32^, in the VTA and basolateral amygdala (BLA) of wildtype mice, respectively, and used a laser with the intensity reduced to 0.5 mW to minimize potential laser-induced damage. Optogenetic stimulation of the VTA-to-NAc projection with reduced laser power still induced a similar inhibition of EPSCs in NAc MSNs, albeit with reduced magnitude (relative EPSCs: 0.90 ± 0.02, p < 0.01, n/m = 61/10; Fig. 2N). In contrast, laser stimulation of the BLA-to-NAc projection did not inhibit electrically-evoked EPSCs in NAcSh MSNs (1.01 ± 0.05, p = 0.79, n/m = 13/3; Fig. 2O). Thus, the optogenetic stimulation-induced short-term inhibition of excitatory synaptic transmission to NAcSh MSNs was primarily attributable to the activation of the VTA-to-NAcSh projection.

**Figure 3:**
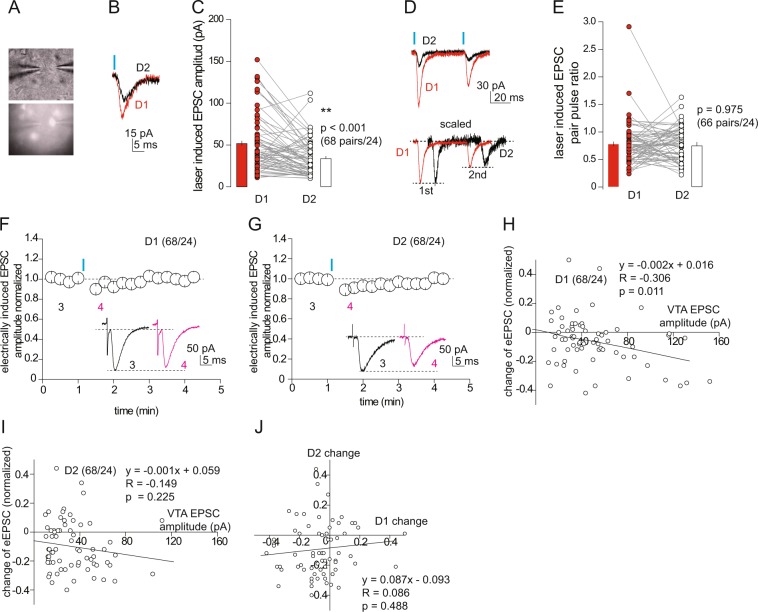
Activation of the VTA-to-NAc projection suppresses EPSCs in both D1 and D2 NAcSh neurons. (**A**) Images showing dual recording, in which two adjacent MSNs were recorded as shown through the DIC channel (upper), and one of them was td-Tomato-positive as shown through the fluorescence channel (lower). Scale bar, 10 µm. (**B**) Example EPSCs recorded in tdTomato-positive D1 and tdTomato-negative D2 NAcSh MSNs evoked by optogenetic stimulation of the VTA-to-NAc projection. (**C**) Summary showing that the mean amplitudes of EPSCs at VTA-to-NAcSh synapses were larger in D1 MSNs compared to D2 MSNs. (**D**) Example EPSCs in D1 and D2 NAcSh MSNs evoked by a paired-pulse optogenetic stimulation protocol. (**E**) Summary showing no statistical difference in the PPR between D1 and D2 MSNs. (**F**,**G**) Summaries and example EPSCs (insets) showing that activation of the VTA-to-NAc projection induced a similar short-term inhibition of electrically-evoked EPSCs in D1 (**F**) and D2 (**G**) NAcSh MSNs. (**H**,**I**) Plots of the magnitudes of short-term inhibition (sampled during the 0–0.5 min period after optogenetic stimulation) against the amplitudes of EPSCs at VTA-to-NAcSh synapses in D1 (**H**) and D2 (**I**) MSNs. (**J**) Plots of the magnitudes of short-term inhibition in paired D1 and D2 MSNs.

D1 and D2 receptor-expressing MSNs are the two major subpopulations of principal neurons in the NAcSh, and are distinctly involved in different aspects of motivated behaviors^5^. To examine whether the modulation mediated by the VTA-to-NAc projection was cell type-specific, we employed a transgenic mouse line in which D1 MSNs are tagged with tdTomato fluorescence^33^. In our electrophysiological recordings, we operationally defined tdTomato-positive and td-Tomato-negative MSNs as D1 versus D2 MSNs, similar to previous studies^34–36^. In addition to the traditional neurotransmitters dopamine and GABA, the VTA projection to the NAcSh releases glutamate and, through monosynaptic connections, evokes excitatory synaptic transmission to both MSNs and interneurons^37–39^. Although the amplitudes of VTA-to-NAc EPSCs do not necessarily predict the magnitudes of release of dopamine, GABA, or other neurotransmitters, it provides a practical measure of the VTA-to-NAcSh connectivity under our current optogenetic setups. We thus sought to directly compare the synaptic strength of the VTA to NAcSh projection onto the two main MSN populations. We performed pairwise comparison by simultaneously recording tdTomato-positive and td-Tomato-negative MSNs upon the same optogenetic stimulation (473-nm, 0.5 mW, 1 ms of pulse duration) of the VTA-to-NAcSh projection. In most dual recordings, stimulation resulted in EPSCs in both D1 and D2 MSNs (Fig. 3A,B). Among the 68 dual recordings from 24 mice, the mean amplitude of the optogenetically-evoked EPSCs was slightly but significantly larger in D1 MSNs compared to D2 MSNs (D1, 50.8 ± 4.0 pA; D2, 33.7 ± 2.7 pA, 68 pairs/24; p < 0.01, paired t-test; Fig. 3C). This observation is consistent with reports that different NAc afferents innervate D1 versus D2 MSNs in a slightly biased manner^7,8^. Despite the overall difference in synaptic strength, the paired pulse ratios (PPRs) of EPSCs evoked by stimulation of the VTA-to-NAcSh projection were similar between D1 versus D2 MSNs, suggesting similar conditions of their presynaptic release machinery onto the two neuronal populations (interpulse interval 50 ms: D1, 0.77 ± 0.05; D2, 0.77 ± 0.04, 66 pairs/24 mice, p = 0.98 D1 vs. D2; Fig. 3D,E).

**Figure 4:**
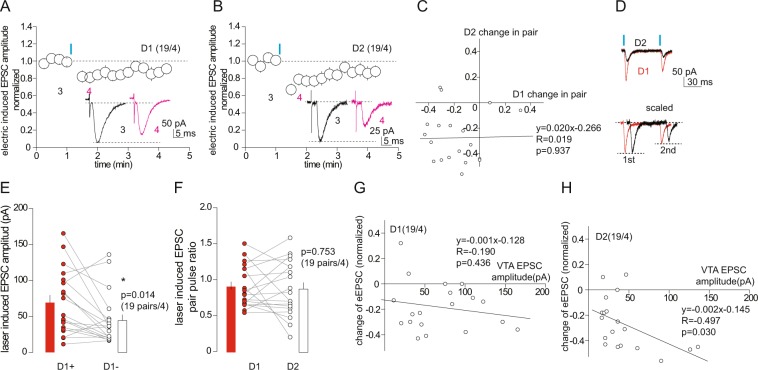
Short-term inhibition of EPSCs by high-power laser-mediated activation of the VTA-to-NAc projection. (**A**,**B**) Summaries and example EPSCS (insets) showing that activation of the VTA-to-NAc projection induced by the laser with increased power (from 0.5 to 1 mW) induced similar short-term inhibition of electrically-evoked EPSCs in both D1 and D2 NAcSh MSNs, but with high magnitudes. (**C**) Plot of dual recordings showing no correlation of the magnitudes of short-term inhibition between D1 and D2 NAcSh MSNs. (**D**) Example EPSCs in D1 and D2 NAcSh MSNs evoked by a paired-pulse optogenetic stimulation protocol. (**E**) Summary showing that the mean amplitudes of EPSCs at VTA-to-NAcSh synapses were larger in D1 MSNs than D2 MSNs in dual recordings. (**F**) Summary showing that the PPR of EPSCs at VTA-to-NAc synapses on D1 and D2 MSNs was similar. (**G**,**H**) Plots of the magnitudes of short-term inhibition against the amplitudes of EPSCs at VTA-to-NAcSh synapses in D1 and D2 MSNs.

Despite the moderate difference in the basal excitatory synaptic strength in the VTA to NAcSh projection, D1 and D2 MSNs exhibited similar short-term inhibition of electrically-evoked EPSCs upon optogenetic activation (0.5 mW) of the ascending VTA-to-NAc projection (relative amplitude after optogenetic stimulation: D1, 0.93 ± 0.02, p = 0.002; D2, 0.90 ± 0.02, p < 0.01, 68 pairs/24, Fig. 3F,G). We detected a weak but significant correlation between the magnitude of inhibition and the amplitude of the optogenetically-evoked EPSCs from VTA-to-NAcSh synapses in D1, but not D2, MSNs (D1, y = −0.002 × + 0.016, R = −0.31, p = 0.01; D2, y = −0.001 × + 0.059, R = −0.15, p = 0.22, Pearson’s correlation test; Fig. 3H,I). Furthermore, the magnitude of this short-term inhibition was not correlated between paired D1 and D2 MSNs (y = 0.087 × −0.093, R = 0.09, p = 0.49, Fig. 3J). When the laser power was increased to 1 mW while keeping other stimulation parameters the same, stimulation of the VTA-to-NAc projection also induced a short-term inhibition of electrically-evoked EPSCs in both NAcSh D1 and D2 MSNs (relative EPSC amplitude after laser stimulation: D1, 0.82 ± 0.05, 19 pairs/4, p = 0.01 1 mW vs. 0.5 mW; D2, 0.73 ± 0.05, 19 pairs/4, p < 0.01 1 mW vs. 0.5 mW; Fig. 4A,B). The magnitude of inhibition (~18% for D1 and 27% for D2) was larger compared to that induced by weaker (0.5 mW) stimulations (~10%) and had a relatively slow recovery time course. Furthermore, the magnitude of inhibition was not correlated between D1 and D2 MSNs in dual recordings (y = 0.020 × −0.266, R = 0.02, p = 0.94, Fig. 4C).

With this relatively stronger optogenetic stimulation (i.e., laser power at 1 mW), our dual recording also detected a biased innervation of D1 over D2 MSNs by VTA-to-NAc excitatory synapses (D1, 69.1 ± 10.6 pA; D2, 44.1 ± 8.4 pA; p = 0.01, paired t-test, 19 pairs/4; Fig. 4D,E). In addition, the VTA-to-NAc excitatory synaptic transmission exhibited a similar PPR between NAcSh D1 versus D2 MSNs under this stimulation protocol, suggesting similar presynaptic release conditions (D1, 0.90 ± 0.07; D2, 0.87 ± 0.07; p = 0.75, paired t-test, 19 pairs/4; Fig. 4F). However, the short-term inhibition induced by the relatively stronger optogenetic stimulation was correlated with the activation intensity of the VTA-to-NAc projection in NAcSh D2, but not D1, MSNs (D1, y = −0.001 × −0.128, R = −0.19, p = 0.44; D2, y = −0.002 × −0.145, R = −0.50, p = 0.03; Fig. 4G,H). Taken together with the correlative results from weak stimulations, it is tempting to speculate that low intensity versus high intensity activation of the VTA-to-NAc projection preferentially regulates D1 versus D2 MSNs.

**Figure 5:**
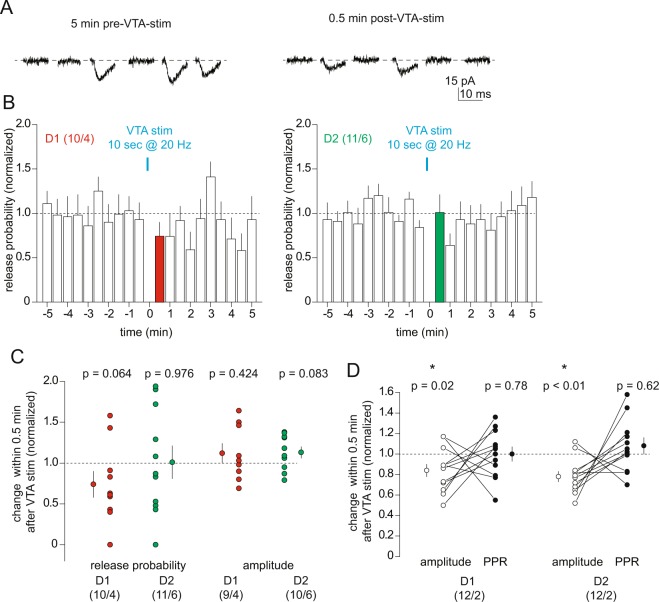
Activation of VTA-to-NAc projection does not affect release probability of excitatory synaptic inputs to NAcSh MSNs. (**A**) Example EPSCs evoked by electrical minimal stimulation before and after optogenetic stimulation of the VTA-to-NAc projection. (**B**) Summaries showing time courses of the relative success rates of minimal stimulation-evoked EPSCs in D1 and D2 NAcSh MSNs before and after optogenetic stimulation of the VTA-to-NAc projection. (**C**) Summary showing that the relative release probability, measured as relative success rates of minimal stimulation-evoked EPSCs, and the relative amplitudes of successful EPSCs were not altered by optogenetic stimulation of the VTA-to-NAc projection. (**D**) Summary showing that the short-term inhibition of electrically-evoked EPSCs in D1 or D2 NAc MSNs was not accompanied by detectable changes in the PPR of EPSCs.

The fact that the short-term inhibition induced by the VTA-to-NAc projection was similarly detected in both NAcSh D1 and D2 MSNs raised the possibility that it was a non-specific effect that would impact all excitatory presynaptic projections to NAcSh MSNs. To address this, we minimized the local presynaptic stimulation intensity such that only a small number of synapses were activated upon a given stimulation. In this case, whether or not each synapse released glutamate was statistically controlled by the presynaptic release probability (Pr), resulting in either successful or failed synaptic transmission (Fig. 5A). As such, the success rate of these small EPSCs over all trials reflects the Pr, while the amplitudes of individual EPSCs reflects the postsynaptic responsiveness.

Using this minimal stimulation strategy, we established the basal EPSCs with relatively stable success rates (operationally defined as relative Pr hereafter) and amplitudes in NAcSh MSNs. We then optogenetically (laser power at 0.5 mW) stimulated the VTA-to-NAc projection, and compared the Pr and amplitudes of EPSCs before and after the stimulation (Fig. 5A,B). Under this condition, optogenetic stimulation of the VTA-to-NAc projection did not affect either the Pr (D1, 0.74 ± 0.16, p = 0.06, 10/4; D2, 1.01 ± 0.20, p = 0.98, 11/6) or the amplitudes (D1, 1.12 ± 0.12, p = 0.42, 9/4; D2, 1.13 ± 0.07, p = 0.08, 10/6) of the EPSCs (Fig. 5C). While these results suggest no presynaptic change, a trend toward inhibition of the Pr was observed in D1 MSNs (Fig. 5C). Further analysis revealed that the magnitude of short-term inhibition in D1 (0.84 ± 0.06, p = 0.02, 12/2) or D2 MSNs (0.78 ± 0.05, p < 0.01, 12/2) was not correlated with any changes in the PPR of the electrically-evoked EPSCs (D1, 1.00 ± 0.07, p = 0.78, 12/2; D2, 1.08 ± 0.08, p = 0.62, 12/2; Fig. 5D). Hence, it is possible that a small presynaptic effect occurred, but it was too subtle to be reliably detected.

**Figure 6:**
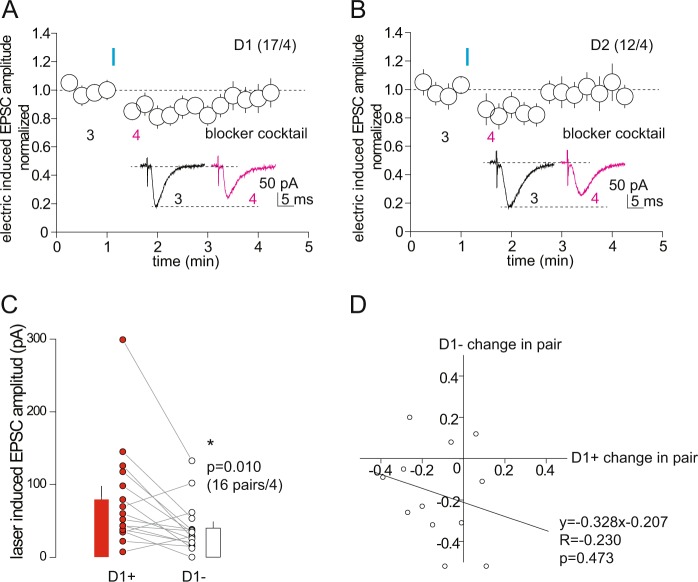
Antagonist cocktail does not prevent activation of VTA-to-NAc projection-induced short-term inhibition of EPSCs in D1 and D2 NAcSh MSNs. (**A**,**B**) Summaries and example EPSCs (insets) showing that, in the presence of antagonist cocktail, containing MRS2179, MTEP, Eticlopride, SCH23390, WAY100638, AM251, D-AP5, CGP55845, Capsazepine, and picrotoxin (all antagonists were present throughout the whole recording procedure), optogenetic stimulation of VTA-to-NAc projection induced short-term inhibition of electrically-evoked EPSCs in D1 (**A**) and D2 (**B**) NAcSh MSNs. (**C**) Summary showing that in the presence of antagonist cocktail, the mean amplitudes of optogenetic stimulation-evoked EPSCs (from VTA-to-NAs synapses) were larger in D1 NAc MSNs compared to their paired D2 MSNs. (**D**) Plot of the magnitudes of short-term inhibition in D2 NAcSh MSNs against paired D1 MSNs.

Activation of the VTA-to-NAc projection results in the releases of a variety of neurotransmitters that can activate pre- or postsynaptically-located receptors to modulate excitatory synaptic transmission to NAcSh MSNs. We examined the possible contribution of some of these VTA-released substances that have been shown to suppress EPSCs in NAc MSNs, including dopamine D1- and D2-class receptors, GABAB receptors, TRP channels, CB1, 5-HT1A receptors, P2Y1 receptors, mGluR5, and NMDA receptors. In the presence of an antagonist cocktail (in μM, 8 MRS2179, 10 MTEP, 8 Eticlopride, 10 SCH23390, 8 WAY100638, 2.5 AM251, 50 D-AP5, 10 CGP55845, 10 Capsazepine; 50 μM GABA_A_ receptor antagonist picrotoxin was included in all recordings), optogenetic stimulation (laser power at 1 mW) of the VTA-to-NAc projection still induced short-term inhibition of electrically-evoked EPSCs in both NAcSh D1 and D2 MSNs (relative EPSC amplitudes after optogenetic stimulation: D1, 0.88 ± 0.04, p = 0.01, 17/4; D2, 0.83 ± 0.07, p = 0.03, 12/4; Fig. 6A,B). In the presence of the antagonist cocktail, the VTA-to-NAc projection once again exhibited biased innervation of D1 over D2 MSNs (D1, 79.4 ± 18.2 pA; D2, 40.2 ± 8.8 pA; p = 0.01, paired t test, 16 pairs/4; Fig. 6C), and the magnitudes of the short-term inhibition in NAcSh D1 and D2 MSNs were not correlated (y = −0.328 × −0.207, R = −0.23, p = 0.47; Fig. 6D). While the contribution of other possible VTA-released substances remains to be examined, these results collectively suggest that the short-term inhibition by VTA-to-NAc activation is not likely mediated by a single or straightforward mechanism.

## Discussion

The VTA-to-NAc projection serves as a key circuit regulating emotional and motivational behaviors. Our current results show that a brief activation of this projection induces a short-term inhibition of the excitatory synaptic transmission to NAcSh D1 and D2 MSNs. This effect, albeit moderate, may transiently and subtly shift the excitation-inhibition balance of MSNs, affecting the overall output of the NAcSh.

NAc D1 versus D2 receptor-bearing MSNs are differentially innervated by glutamatergic projections from several major limbic regions^7,8^. Here we show a similar differential innervation of D1 and D2 MSNs from the VTA glutamatergic projection. A predicted consequence of these biased innervations is that upon the input of emotional and motivational arousal signals, NAc D1 MSNs receive overall stronger excitatory driving force compared to D2 MSNs. However, compared to NAc D2 MSNs, D1 MSNs exhibit substantially lower intrinsic membrane excitability^40^, a parameter reflecting the ability of neurons to generate action potentials in response to excitatory input. As such, the actual excitation patterns of NAc D1 versus D2 MSNs as measured by action potential firing in response to emotional and motivational arousals rely on the dynamic integration of synaptic inputs and membrane properties. This idea is supported by *in vivo* imaging results showing dynamic excitation of NAc D1 and D2 MSNs in motivated behaviors^6^.

For goal-directed behaviors, dopaminergic neurons, GABAergic neurons, and possibly other neuronal types in the VTA are often activated in the same time blocks, releasing a large number of neurotransmitters and biologically active factors to the NAc that regulate behavioral initiation and performance. Many of these VTA-induced substances, including dopamine, GABA, glutamate, ATP, and endocannabinoids, have been examined independently for their modulation of NAc excitatory synaptic transmission. In each case, alterations in evoked EPSCs have been measured in response to slice perfusion of the substance itself or an agonist of its receptor. Under these experimental conditions, most of these neurotransmitters or their coupled receptor signaling pathways have been shown to suppress the amplitudes of evoked EPSCs in NAc MSNs, often with high inhibition magnitudes^41–46^. In our experimental setup, however, the effects of VTA activation were rather moderate.

At least two ideas are worth considering when interpreting these results. First, the procedures in which these substances or related agonists were perfused in slices often took a time range of several minutes, while the endogenous release of these substances occurs within a time range of milliseconds to seconds. Thus, the synaptic effects detected by prolonged receptor activation may result from sustained changes that do not normally occur after phasic activation of the VTA as better mimicked by our optogenetic procedures. Second, most prior studies that used the drug perfusion approach focused on a single substance each time, while optogenetic activation of the VTA-to-NAc projection most likely released a large number of substances simultaneously. These substances might then interact at the receptor and signaling levels to reach a combined effect on synaptic transmission.

Despite the fact we simultaneously inhibited nine different receptors of VTA substances, the short-term inhibition induced by activation of the VTA-to-NAcSh projection remained intact. Given that we did not inhibit an exhaustive list of VTA-released substances, one of our hypothesized scenarios is that this short-term inhibition might be mediated by other synaptic modulators that are directly released from the VTA-to-NAc projection or from other NAc neuronal populations that are activated by the VTA-to-NAc projection. For example, even though dense-core vesicles are infrequently observed in this projection, it would be prudent in future studies to examine the possible role of peptide transmitters, such as neurotensin or cholecystokinin^25,26^. Another hypothesized scenario is that the VTA-to-NAc projection activates astrocytes, with the resulting change in astrocytic signaling altering excitatory synaptic transmission to MSNs. Indeed, axons from the VTA are often seen in contact with astrocytes, and in the spinal cord purines released from astrocytes acutely inhibit excitatory synaptic transmission^47^.

Beyond receptor-mediated modulation, the short-term inhibition might also have stemmed from a biophysical effect. For example, VTA-to-NAc projections might be uniquely aligned with excitatory synapses on NAcSh MSNs such that the resulting depolarization, neurotransmitter release, or changes of ionic environment could induce a desensitization of the postsynaptic membrane that resulted in transient inhibition. Although the electron microscopic details of the VTA projection to NAc interneurons have been examined^38^, the VTA innervation of MSNs, and more critically, the anatomical relationships between VTA-originated synapses and other excitatory synapses on NAc MSNs remain underexplored.

In summary, our current results depict a new form of short-term plasticity at excitatory synapses on NAcSh MSNs induced by the VTA-to-NAc projection. This short-term suppression may transiently change the excitation-inhibition balance of NAc MSNs during motivated behaviors, during which the VTA-to-NAc projection is activated in a brief and bursting manner.

## Materials and Methods

### Animals

Both male and female C57BL/6J wild type mice, TH-IRES-Cre mice, and Drd1a-tdTomato transgenic mice (Jackson Laboratory) at the age of 6–10 weeks old were used in all experiments. Mice were singly housed on a regular 12-h light/dark cycle (light on/off at 7:00 AM/PM), with food and water available ad libitum. The animals were used in accordance with the NIH guideline of experimental animal use and under protocols approved by the Institutional Animal Care and Use Committees at University of Pittsburgh.

### Electron microscopy

Tract-tracing was performed in 6 wild type male mice at postnatal days of 60–80. Four to five weeks after the intra-VTA injection of eGFP, mice were anesthetized with 60 mg/kg pentobarbital, i.p. and then treated for 15 min with 1 g/kg i.p. of the zinc chelator, diethyldithiocarbamic acid to prevent artifactual silver labeling of endogenous zinc^48^. Mice were then perfused through the aorta with 5–10 ml of heparin-saline 100 U/ml, followed by 12.5 ml of 3.75% acrolein in 2% paraformaldehyde, followed by 75 ml of 2% paraformaldehyde. The fixative was made in 0.1 M phosphate buffer, pH 7.4 (PB). Blocks of fixed brain containing the NAc and VTA were cut on a vibratome at 50 µm, and tissue sections were collected in PB. Sections were treated for 30 min in 1% sodium borohydride in PB and then rinsed extensively in PB. Sections were then transferred to 0.1 M tris-buffered saline, pH 7.6 (TBS) before being incubated for 30 min in a blocking solution containing 1% bovine serum albumin, 3% normal goat serum and 0.04% Triton X-100 in TBS (0.2% Triton was used for light microscopic tissue).

Brain sections were incubated over 1–2 nights in primary antibody, which included either chicken anti-GFP (Avēs Labs, GFP-1010) at 1:500–1:1000 for single labeling (1 night, 4 mice) or a mixture of anti-GFP at 1:1000 and an antibody raised in guinea pig against vGlut2 (Millipore/Chemicon, AB2251) at 1:1000 for double labeling (2 nights, 2 mice). In some cases, sections were placed overnight in the vGlut2 antibody before the GFP antibody was added 24 hours later. Sections were then rinsed extensively in TBS to remove unbound antibody. For double labeling, the presence of bound vGlut2 antibody was revealed first using the standard avidin-biotin peroxidase procedure and diaminobenzidine as the substrate. The presence of bound eGFP (single or double labeling) was visualized by silver-enhanced immunogold as follows. Sections were rinsed in 0.01 M phosphate buffered saline, pH 7.4 (PBS) and then placed in a washing buffer containing 3% normal goat serum, 0.8% bovine serum albumin, and 0.1% cold fish gelatin. Tissue was then transferred to a secondary antibody conjugated to 0.8 nm gold particles (Aurion) and incubated at 1:50 in washing buffer overnight (12–15 hrs). After rinsing in washing buffer and then PBS, sections were incubated in 2.5% glutaraldehyde in PBS for 10 min and rinsed again in PBS. The tissue was then placed in Enhancement Conditioning Solution (ECS; Aurion) diluted 1:10 with ultrapure water for four 10 min exchanges. Tissue was then incubated in RGENT-SEM proprietary silver solution (Aurion) for ~120 min and rinsed again for four 10 min exchanges in ECS. Sections were then transferred to PB for electron microscopy tissue processing.

In addition to these tissue sets, one set of sections through the NAc and VTA was processed by single immunoperoxidase labeling of eGFP for light microscopic verification of injection sites and anterograde transport. In one mouse, the aldehyde perfusion was considered inadequate for electron microscopic tissue preservation, and only light microscopic sections were processed. This left 3 mice for single labeling of GFP and 2 mice for dual labeling of GFP and vGlut2.

Tissue preparation for electron microscopy included lipid fixation with osmium tetroxide at 1% in PB for 1 hr, followed by rinsing in PB. Sections were then dehydrated through increasing concentrations of ethanol in PB followed by propylene oxide. Sections were then transferred to a 1:1 mixture of propylene oxide and epoxy resin (EMBed 812, Electron Microscopy Sciences) and incubated overnight. The following day, tissue was transferred to straight epoxy resin for 2–3 hours. The sections were then carefully laid onto commercial plastic film, coverslipped with an additional plastic sheet, put under heavy weights to keep the tissue flat, and then placed in a 60 °C oven until the resin was cured.

The NAcSh was identified in resin-cured brain sections, and a small part of this region was trimmed to a trapezoid shape using razor blades. Ultrathin sections at 60 nm were then cut from the surface of these sections and collected onto copper 400 mesh grids. The grids were stained with uranyl acetate and lead citrate to add contrast to the tissue, and the tissue was examined in an FEI Morgagni transmission electron microscope. Random sampling was used to identify neuronal structures throughout the NAcSh that contained single immunogold-silver labeling for transported eGFP or dual immunogold for eGFP and immunoperoxidase for vGlut2.

### Viral vectors and drugs

AAV2-hSyn-hChR2(H134R)-EYFP and AAV8-Syn-ChrimsonR-tdTomato were from the University of North Carolina viral vector core.

MTEP, Eticlopride, WAY 100635, D-AP5, and CGP 55845 were purchased from Tocris. SCH 23390 was purchased from Cayman Chemical. AM251 was purchased from APExBIO. Capsazepine was purchased from Toronto Research Chemicals.

### Viral Delivery

Mice were anesthetized with a ketamine (50 mg/kg)–xylazine (5 mg/kg) mixture (i.p.). A 33-gauge needle was used to bilaterally inject 0.7 μL/site (0.33 μL/min) of the AAV2-hSyn-hChR2(H134R)-EYFP solution into the VTA (to lambda, in millimeters, AP, +1.35; ML, ±0.38; DV, −4.60), or AAV8-Syn-ChrimsonR-tdTomato into BLA (to bregma, AP, −1.00; ML, ±3.00; DV, −5.00). After surgery, mice were place on a heating pad for recovery. Carprofen (5 mg/kg) was injected (s.c.) daily for up to 3 days after surgery. Mice were kept in their home cages for 4 to 5 weeks for viral expression before electrophysiological experiments.

### Preparation of NAc slices

Mice were decapitated following isoflurane anesthesia. Sagittal slices (250 μm) containing the NAc were prepared on a VT1200S vibratome (Leica) in 4 °C cutting solution containing (in mM) 135 N-methyl-d-glucamine, 1 KCl, 1.2 KH_2_PO_4_, 0.5 CaCl_2_, 1.5 MgCl_2_, 20 choline-HCO_3_, and 11 glucose, saturated with 95% O_2_/5% CO_2_, pH adjusted to 7.4 with HCl. Osmolality was adjusted to 300 mOsm/l. Slices were then held in artificial cerebrospinal fluid (aCSF) containing (in mM) 119 NaCl, 2.5 KCl, 2.5 CaCl_2_, 1.3 MgCl_2_, 1 NaH_2_PO_4_, 26.2 NaHCO_3_, and 11 glucose, with the osmolality adjusted to 290. aCSF was saturated with 95% O_2_/5% CO_2_ at 34 °C for 30 min. The slice holding container was then moved into another chamber at 20–22 °C > 30 min before the slices were used for experimentation. This preparation procedure has been verified and routinely used in our previous studies^37,49,50^.

### Electrophysiological recordings

All recordings were made in the medial NAcSh. Dual recordings were made from two adjacent neurons <50 μm apart. To record EPSCs in MSNs, electrodes were filled with an internal solution containing (in mM) 130 KMeSO_4_, 10 KCl, 10 Hepes, 0.4 EGTA, 3 Mg-ATP, 0.5 Na_3_-GTP, 2 MgCl_2_, 7.5 phosphocreatine.

The series resistance was typically within the 9–20 MΩ range, uncompensated, and monitored continuously during recording. Recorded neurons with a change in series resistance >15% were not accepted for data analysis. Synaptic currents were recorded with a MultiClamp 700B amplifier (Molecular Devices), filtered at 2.6–3 kHz, amplified five times, and then digitized at 20 kHz. Optogenetic stimulation was achieved using a laser with the wavelength of 473 nm (IkeCool) or 635 nm (Laser Century). Stimulation parameters were preprogrammed using Clampex software (Molecular Devices). Collimated laser light was coupled to a fluorescent port of the Olympus BX51WI microscope, allowing the laser to illuminate the recorded slice. The laser intensity through the lens was measured by a light sensor (S130A; Thorn Labs) before recordings. The pulse duration was fixed at 1.0 ms. In recording of optogenetically evoked EPSCs, a two-pulse stimulation was delivered with a 50-ms interval over a single trial. For optogenetically evoked burst stimulation, pulse duration and interval remained unchanged. Only one burst was induced in each neuron unless otherwise stated. For electrically induced EPSCs, stimulation was delivered every 7.5 sec. For minimal stimulation experiments, electric stimulation was delivered every 5 sec.

### Data acquisition and statistics

All data were analyzed offline. We performed the Kolmogorov-Smirnov test for main data sets, all of which were normally distributed, including data in Fig. 2N (p = 0.99), Fig. 3F (p = 0.45), Fig. 3G (p = 0.16), Fig. 4A (p = 0.82), and Fig. 4B (p = 0.77). Samples in the statistical analysis were individual neurons or neuron pairs. To compare the EPSC amplitudes before and after optogenetic stimulation of the VTA projection, we averaged the amplitudes of all five EPSCs before the optogenetic stimulation as the baseline mean, and the five EPSCs over the 30-sec period right after optogenetic stimulation as the mean after manipulation. We then performed t-tests to compare these two sets of data. Sample size was presented as n/m, representing that “n” cells used in the statistics were from “m” mice. Statistical resultswere expressed as mean ± SEM. Two-tailed t tests were used for statistical comparisons.
